# Delineating the elusive BaMMV resistance gene *rym15* in barley by medium-resolution mapping

**DOI:** 10.1007/s11032-021-01270-9

**Published:** 2021-12-02

**Authors:** Yaping Wang, Antje Habekuß, Rod J. Snowdon, Frank Ordon, Dragan Perovic

**Affiliations:** 1 Institute for Resistance Research and Stress Tolerance, Federal Research Centre for Cultivated Plants, Julius Kuehn-Institute, Erwin-Baur-Strasse 27, 06484 Quedlinburg, Germany; 2 Department of Plant Breeding, IFZ Research Centre for Biosystems, Land Use and Nutrition, Justus Liebig University, Giessen, Germany

**Keywords:** Barley, BaMMV mechanical inoculation, *rym15*, Medium-resolution mapping, SSR, KASP

## Abstract

*Barley mild mosaic virus* (BaMMV), transmitted by the soil-borne protist *Polymyxa graminis*, has a serious impact on winter barley production. Previously, the BaMMV resistance gene *rym15* was mapped on chromosome 6HS, but the order of flanking markers was non-collinear between different maps. To resolve the position of the flanking markers and to enable map-based cloning of *rym15*, two medium-resolution mapping populations Igri (susceptible) × Chikurin Ibaraki 1 (resistant) (I × C) and Chikurin Ibaraki 1 × Uschi (susceptible) (C × U), consisting of 342 and 180 F_2_ plants, respectively, were developed. Efficiency of the mechanical inoculation of susceptible standards varied from 87.5 to 100% and in F_2_ populations from 90.56 to 93.23%. Phenotyping of F_2_ plants and corresponding F_3_ families revealed segregation ratios of 250 s:92r (I × C, *χ*^2^ = 0.659) and 140 s:40r (C × U, *χ*^2^ = 0.741), suggesting the presence of a single recessive resistance gene. After screening the parents with the 50 K Infinium chip and anchoring corresponding SNPs to the barley reference genome, 8 KASP assays were developed and used to remap the gene. Newly constructed maps revealed a collinear order of markers, thereby allowing the identification of high throughput flanking markers. This study demonstrates how construction of medium-resolution mapping populations in combination with robust phenotyping can efficiently resolve conflicting marker ordering and reduce the size of the target interval. In the reference genome era and genome-wide genotyping era, medium-resolution mapping will help accelerate candidate gene identification for traits where phenotyping is difficult.

**Supplementary Information:**

The online version contains supplementary material available at 10.1007/s11032-021-01270-9.

## Introduction

Barley yellow mosaic disease is caused by two related viruses, *barley yellow mosaic virus* (BaYMV) and *barley mild mosaic virus* (BaMMV). The disease can heavily impact winter barley cropping, with 40–80% yield loss in 2-rowed barley in Japan (Usugi 1988; Ohto 2000), and 50% losses in Europe (Plumb et al. 1986; Adams et al. 1992; Overthrow et al. 1999) up to complete yield loss, e.g., in some counties of the Yangtze River Valley (Chen 1993, 2005; Chen and Ruan 1992). Both viruses belong to the genus *Bymovirus* in the family *Potyviridae* and are transmitted by the root-infecting plasmodiophorid *Polymyxa graminis* L. However, the two causal viruses differ in their temperature optima, serological properties, and transcriptomes and their ability to infect different barley genotypes (Huth and Adams 1990; Habekuß et al. 2008). Use of resistant cultivars is the most economical and environmentally friendly way to control these soil-borne viruses (Kanyuka et al. 2003). So far, 22 resistance genes against barley yellow mosaic disease have been reported, of which most are recessive genes (see review of Jiang et al. 2020). However, many of these resistance genes are no longer effective. For example, the resistance gene *rym4* is ineffective against BaYMV-2, which appeared in the late 1980s; the resistance gene *rym5* was overcome by the strain BaMMV-Sil in France and BaMMV-Teik in Germany (Hariri et al. 2003; Vaianopoulos et al. 2007; Habekuß et al. 2008). It may therefore be expected that this trend will continue in the future; based on this, it is essential to identify and further characterize new sources of resistance and to develop diagnostic markers for marker-assisted selection (MAS) in barley.

About half of the known virus resistance genes in crops are recessive (Kang et al. 2005; Robaglia and Caranta 2006; Wang and Krishnaswamy 2012). Plant viruses need to recruit the host cells’ machinery to complete the infectious life cycle; thus, mutation in the host factors genes may result in virus resistance (Garcia-Ruiz, 2018). Several of these recessive resistance genes are isoforms of *eukaryotic translation initiation factor 4E* (*eIF4E*), and *eIF4G* (Moffett 2009; Hashimoto et al. 2016). Up to now, two recessive resistance genes against BaMMV/BaYMV in barley have been isolated. The resistance to BaMMV/BaYMV impacted by the *rym4*/*5* locus is due to the host factor gene *HvEIF4E* (Kanyuka et al. 2005; Stein et al. 2005), while *rym1*/*11* resistance is caused by sequence variations of the host factor gene *Protein Disulfide Isomerase Like 5*–*1* (*HvPDIL5*-*1*) (Yang et al. 2014a). Out of twenty-two reported BaMMV/BaYMV resistance genes, six are allelic forms of *HvEIF4E*, i.e., *rym4*, *rym5*, *rym6*, *rym10*, *eIF4E*_HOR4224_, and *eIF4E*_HOR3298_, while two (*rym1* and *rym11*) are allelic forms of *HvPDIL51* (Perovic et al. 2014; Yang et al. 2014a; Shi et al. 2019).

The Japanese barley landrace Chikurin Ibaraki 1 is susceptible to BaYMV in Japan (Ukai and Yamashita 1980). In contrast to this, Chikurin Ibaraki 1 was found to be resistant in response to three European strains, i.e., BaMMV, BaYMV-1, and BaYMV-2 (Götz and Friedt 1993; Lapierre and Signoret 2004). Werner et al. (2003) demonstrated that an uncharacterized recessive resistance locus on chromosome 5HS effective against BaYMV and BaYMV-2 originates from Chikurin Ibaraki 1 and segregates independently from the Carola-derived *rym4* resistance that is effective against BaYMV and BaMMV. Further analysis of a doubled haploid (DH) mapping population derived from the cross of the Chikurin Ibaraki 1 and the susceptible winter barley cv. Plaisant located the recessive resistance gene effective against BaMMV on the short arm of chromosome 6H that was subsequently named *rym15* (Le Gouis et al. 2004). However, the study showed that the order of flanking markers EBmac0874 and Bmag0173 is inverted compared to the genetic map of Lina × *Hordeum spontaneum* Canada Park (Ramsay et al. 2000). To date, this discrepancy in the marker order spanning the resistance locus has hindered further map-based cloning efforts for *rym15*.

During BaMMV/BaYMV testing in fields, there are many obstacles, e.g., an uneven distribution of the virus, simultaneous occurrence of two viruses (BaMMV and BaYMV), and similarity of the symptoms (Huth et al. 1984). In addition, only 1 cycle of winter barley resistance testing per year highlights the demand for a reliable and efficient testing method of soil-borne viruses of barley. Consequently, the mechanical inoculation method could overcome the variation in year-to-year scoring of the resistance reaction from the same genotype in the same field that is due to the abovementioned variable environmental factors (Friedt 1983; Pandey 2006). Up to now, several mechanical inoculation methods for BaMMV were developed, e.g., based on soaked sponge rubbing (Friedt 1983), airbrush (Adams et al. 1986), finger rubbing (Kashiwazaki et al. 1989; Habekuß et al. 2008), spray gun (Ordon and Friedt 1993), or stick with gauze (SWG) methods (Jonson et al. 2006). Those studies suggested that the additives, the inoculation stages, the temperatures, and the inoculation techniques of the virus might influence the inoculation efficiency. While BaMMV is readily transmissible, the efficiency of BaYMV is much lower and is usually below 50% (So et al. 1997). Therefore, the knowledge of various degrees of mechanical inoculation efficiency should be taken in account for optimization of map-based cloning projects.

In the past 25 years, molecular markers have been increasingly used in the genetic analysis of various traits and nowadays have become the basic tool for effective mapping of resistance genes in all crop plant species (Garrido-Cardenas et al. 2018; Perovic et al. 2019). Various codominant marker platforms have been used effectively to map resistance genes in crop plants. Simple sequence repeat (SSR) markers or microsatellites are highly polymorphic and reproducible; however, they are not amenable for high throughput even in the case of modified capillary systems (Perovic et al. 2013a) nor as abundant as single-nucleotide polymorphism (SNP). Due to the property of abundance and high throughput, SNP markers have become the most amenable for gene mapping and breeding (Silvar et al 2011; Rasheed et al. 2017; Lu et al. 2020).

In case of barley, SNP arrays (Comadran et al. 2012; Bayer et al. 2017) provide the accurate physical marker position based on the most recent reference genome assembly data (Mascher et al. 2017; Monat et al. 2019). This feature greatly enhances the efficiency of breeding and genetic studies in barley (Perovic et al. 2020). Based on the published barley reference sequence (Mascher et al. 2017) and exome capture data (Russell et al. 2016), the 50 K Illumina Infinium genotyping array was developed, featuring 49,267 SNP markers that were converted into 44,040 working assays (Bayer et al. 2017). Compared with the 9 K Infinium iSelect array, which contained 7842 markers (Comadran et al. 2012), the 50 K Illumina Infinium array possesses around six times more markers, resulting in cheaper genotyping costs per sample.

The main objectives of the present study were to construct two medium-resolution maps for the BaMMV resistance gene *rym15*, resolve the discrepancy in the order of flanking markers, and develop robust high-throughput amenable flanking markers as a prerequisite for map-based cloning of the resistance gene *rym15*.

## Material and methods

### Plant material

The resistant Japanese cultivar Chikurin Ibaraki 1 was crossed with the susceptible cultivars Igri and Uschi. A set of 342 and 180 F_2_ plants derived from the crosses Igri × Chikurin Ibaraki 1 (I × C) and Chikurin Ibaraki 1 × Uschi (C × U) was used to construct the medium-resolution maps for BaMMV resistance gene *rym15* (Supplementary Table 1). In addition, F_3_ progeny was used for the validation of phenotypic data (Supplementary Table 2).

### Resistance test

A set of 522 F_2_ plants and corresponding F_3_ families (Supplementary Table 2) was mechanically inoculated by the isolate BaMMV-ASL, and the crossing parents (Chikurin Ibaraki 1, Igri, and Uschi) and Maris Otter (positive control) were included in all 15 batches of the phenotypic analysis. After sowing, the plants were cultivated in the greenhouse for 7 days followed by cultivation in a growth chamber at 12 °C, 70% relative humidity, and 16-h (14:00–6:00) photoperiod (illuminance 20 klux). The mechanical inoculation was conducted according Perovic et al. (2014) with minor changes. All plant samples were inoculated at the 2- to 3-leaf stage two times at an interval of 5–7 days using sap extracted from the leaves of infected Maris Otter by homogenization in 0.1 M K_2_HPO_4_ buffer, pH 9.8. Approximately, 0.2 mL of buffer was used for each 1 g of infected leaf material. To aid mechanical inoculation, 0.1 g of carborundum (mesh 400) was added per 1 mL sap. Six weeks after the first inoculation, the leaves of tested plants were sampled and the double antibody sandwich ELISA (DAS-ELISA) was carried out according to Clark and Adams (1977) using polyclonal antibodies prepared at JKI (Quedlinburg, Germany). Virus particles were estimated via extinction at 405 nm using a Dynatech MR 5000 microtiter-plate reader at 30 min and 60 min after addition of p-nitrophenyl phosphate (PNPP) substrate buffer. All F_2_ and F_3_ plants with an extinction E405 > 0.1 were qualitatively scored as susceptible.

Based on the phenotypic data of susceptible parental lines Igri, Uschi, and positive control Maris Otter, the success rate of the mechanical inoculation method firstly was calculated by dividing the number of ELISA-positive susceptible plants with the total number of inoculated ones of these three genotypes. To evaluate the inoculation efficiency in the populations, the genotypic data of susceptible F_2_ (homozygous/heterozygous) and F_3_ (homozygous) plants were compared with the phenotypic data; the efficiency was calculated using the following equation:

$$\mathrm{BaMMV}\;\mathrm{inoculation}\;\mathrm{efficiency}=\frac{\mathrm{number}\;\mathrm{of}\;\mathrm{susceptible}\;\mathrm{plants}\;(\mathrm{based}\;\mathrm{on}\;\mathrm{ELISA}\;\mathrm{scores})}{\mathrm{total}\;\mathrm{number}\;\mathrm{of}\;\mathrm{plants}\;\mathrm{analysed}\;\mathrm{for}\;\mathrm{BaMMV}\;(\mathrm{based}\;\mathrm{on}\;\mathrm{marker}\;\mathrm{analysis})}\times100\%$$

### DNA extraction and SSR marker analysis

In order to make the genetic analysis by SSR markers for all F_2_ plants and parental lines Chikurin Ibaraki 1, Igri, and Uschi, DNA was extracted from barley seedlings of 14 days old using CTAB (cetyltrimethylammonium bromide) method according to Stein et al. (2001). The concentration and quality of DNA were estimated using the NanoDrop ND-1000 spectrophotometer (PeQLab, Erlangen, Germany). A set of six SSR markers linked to *rym15* (Bmac0127, Bmac0018, Bmag0867, Bmag0870, EBmac0874, and Bmag0173; Le Gouis et al. 2004) was chosen for genotyping the parental lines and 522 F_2_ plants. PCR reaction consisting of 1 μL of template DNA (25–30 ng/μL), 1 μL of 10 × buffer, 1 μL of 25 mM MgCl_2_, 0.2 μL of 10 mM dNTP-Mix, 0.25 μL of each forward primer (10.0 pmol/μL) and reverse primer (10.0 pmol/μL), and 0.08 μL of 5 U HOT FIREPol DNA polymerase (Solis BioDyne, Tartu, Estonia). M13-tails were added to the forward primers, for SSR amplification, so that 0.1 μL of M13 primer (10.0 pmol/μL) (5′-CAC GAC GTT GTA AAA CGA C-3′) labeled with 5′ fluorescent dyes was added to the reaction mix in a final volume of 10 μL (Macdonald et al. 2006; Perovic et al. 2013b). DNA was amplified in a GeneAmp PCR System 9700 (Applied Biosystems) for all SSR markers under the following conditions: 94 °C for 5 min; followed by touchdown PCR with 12 cycles of 30 s at 94 °C, 30 s at 62 °C, 30 s at 72 °C, and then 35 cycles with 30 s at 94 °C, 30 s at 56 °C, and 30 s at 72 °C, and a final extension at 72 °C for 10 min. Amplified products (1 μL) were checked on an agarose gel (1.5%). For the capillary-based scoring, 1 μL of the PCR product was mixed with Hi-Di™ formamide (Applied Biosystems) and GeneScan™-500 ROX™ size standard (Applied Biosystems) (0.03 μL ROX: 14 μL HiDi™ formamide). The mixture was then denatured for 5 min at 94 °C and subjected to capillary electrophoresis in an ABI PRISM 3100 genetic analyzer (Applied Biosystems). Data was collected using 3130xl data collection software v3.0 (Applied Biosystems). The size of the detected alleles was determined using the GeneMapper v4.0 (Applied Biosystems).

The physical position of the SSR markers was determined by blasting forward and reverse primers against the barley reference genome sequence (http://webblast.ipk-gatersleben.de/barley_ibsc/) using default parameters of blastN.

### 50 K Illumina Infinium genotyping array and KASP marker development

In order to identify polymorphisms between parental lines (Chikurin Ibaraki 1, Igri, and Uschi) and develop markers for genetic analysis for both populations, the DNA of three parental lines (Chikurin Ibaraki 1, Igri, and Uschi) was analyzed by using the 50 K Illumina Infinium genotyping array at the company TraitGenetics (Gatersleben, Germany). The additional information (locus name, position, and sequence) on 50 K array SNPs was downloaded from iSelect (http://bioinf.hutton.ac.uk/iselect/app/). The SNP dataset was filtered using Excel software; on each chromosome, the homozygous SNPs between Chikurin Ibaraki 1 and Igri were identified and the same analysis was conducted for Chikurin Ibaraki 1 and Uschi. Based on the Infinium 50 K data, a set of eight SNPs was selected for the design of competitive allele-specific PCR (KASP) assays (*rym15*_1, *rym15*_4, *rym15*_6, *rym15*_8, *rym15*_11, *rym15*_13, *rym15*_15, *rym15*_17, Supplementary Table 3) by using the website BatchPrimer3 (You et al. 2008); the parameter of product size is 70–150 base pair. All eight KASPs were used to genotype the three parental lines and 522 F_2_ plants. The PCR reaction consisted of 2.2 μL of template DNA (25–30 ng/μL), 0.2 μL of common primer (10.0 pmol/μL), 0.08 μL of each allele-specific primer 1 and allele-specific primer 2 (10.0 pmol/μL), and 2.5 μL of 2 × KASP Master Mix. DNA was amplified in the CFX96 Touch Real-Time PCR Detection System (Bio-Rad) with the following conditions: 94 °C for 15 min; followed by PCR with 9 cycles (− 0.6 °C/cycle) of 20 s at 94 °C, 1 min at 61 °C, and then 25 cycles with 20 s at 94 °C, 1 min at 55 °C, and a final cool down at 30 °C for 1 min. If necessary, recycling with the following conditions was performed: 94 °C for 3 min, followed by PCR with 9 cycles of 20 s at 94 °C, 1 min at 57 °C, and a final cool down at 30 °C for 1 min. The fluorescence signals from HEX and FAM for the specific alleles were detected using the same Detection System (Bio-Rad) at 37 °C after thermal cycling was complete. At the end of the run, the results were displayed in the data analysis software under “Allelic Discrimination” (LGC, Guide to running KASP genotyping on the Bio-Rad CFX-series instruments).

### Linkage analysis

The observed segregation ratios of F_1:2_ (1:3) and F_2:3_ (1:2:1) for the inheritance of a single recessive gene were tested using chi-squared (*χ*^2^). Based on the genotypic and verified phenotypic data of all F_2_ plants, the genetic maps were constructed using the software JoinMap v.4 (Van Ooijen 2006) applying the Kosambi function (Kosambi 1944) and a LOD score of 3.

## Results

### Phenotypic analysis

A set of 522 F_2_ plants was mechanically inoculated using BaMMV-ASL isolate. In order to test integrity of individual F_2_ plants, the phenotypic analysis of corresponding F_3_ families was conducted (Supplementary Table 4). Based on phenotyping of the F_2_ and F_2:3_ generations, 342 (I × C) and 180 (C × U) F_2_ plants showed the segregation of 250 s:92r (*χ*^2^ = 0.659) and 140 s:40r (*χ*^2^ = 0.741), respectively. Chi-square test indicated that these ratios fit to a 3 s:1r segregation ratio (Supplementary Table 4). In the F_3_ generation, the ratio of non-segregating susceptible to segregating susceptible to resistant F_2:3_ plants from I × C and C × U was 74:176:92 (*χ*^2^ = 2.187) and 53:87:40 (*χ*^2^ = 2.078), respectively. Chi-square test indicated that these ratios fit to a 1:2:1 segregation ratio (Supplementary Table 4). Overall, the resistance data of F_2_ populations I × C and C × U suggest a single recessive gene causing resistance against BaMMV in Chikurin Ibaraki 1.

The entire phenotypic analysis of all F_2_ plants and corresponding F_3_ families was accomplished in 15 batches due to the space and time constraints in the growth chamber. Regarding analysis of susceptible control genotype, out of 204 DAS-ELISA-analyzed Maris Otter plants, seven escaped from the virus inoculation. At the same time, for the parental line Igri, five out of 40 inoculated ones escaped, while all of 36 Uschi plants were successfully inoculated. Based on these data, the inoculation rates in the susceptible control Maris Otter as well as the susceptible parental lines Igri and Uschi are 96.35%, 87.5%, and 100%, respectively. In the populations I × C, 16 false positives and 13 escapes were identified among the F_2_ plants, while in the population C × U, nine plants were false positive and 13 escaped (Supplementary Table 5). Accordingly, 29 (8.47%, I × C) and 22 (12.22%, C × U) F_2_ plants with the deduced F_2_ phenotypic data based on F_2:3_ phenotyping analysis were used for further linkage analysis. Based on all phenotypic data of the susceptible F_2_ (homozygous/heterozygous/recombinant) and F_3_ (homozygous) plants in the populations I × C and C × U, the efficiency of inoculation method varied from 90.56 to 93.23% (Table 1).

**Table 1:** Efficiency of the mechanical inoculation method of BaMMV

F_2_ population	Heterozygous susceptible F_2_	Homozygous susceptible F_2_	Recombinant susceptible F_2_	Homozygous susceptible F_3_	Total	Efficiency
Igri × Chikurin Ibaraki 1	161^a^	74^a^	7^a^	150^a^	329^a^	90.56%
	151^b^	72^b^	6^b^	126^b^	355^b^	
Chikurin Ibaraki 1 × Uschi	79^a^	50^a^	7^a^	130^a^	266^a^	93.23%
	70^b^	47b	6^b^	125^b^	248^b^	

^a^The number of different types of susceptible plants based on the genotypic analysis

^b^The number of susceptible plants based on phenotypic analysis

### Molecular marker genotyping

**Fig. 1:**
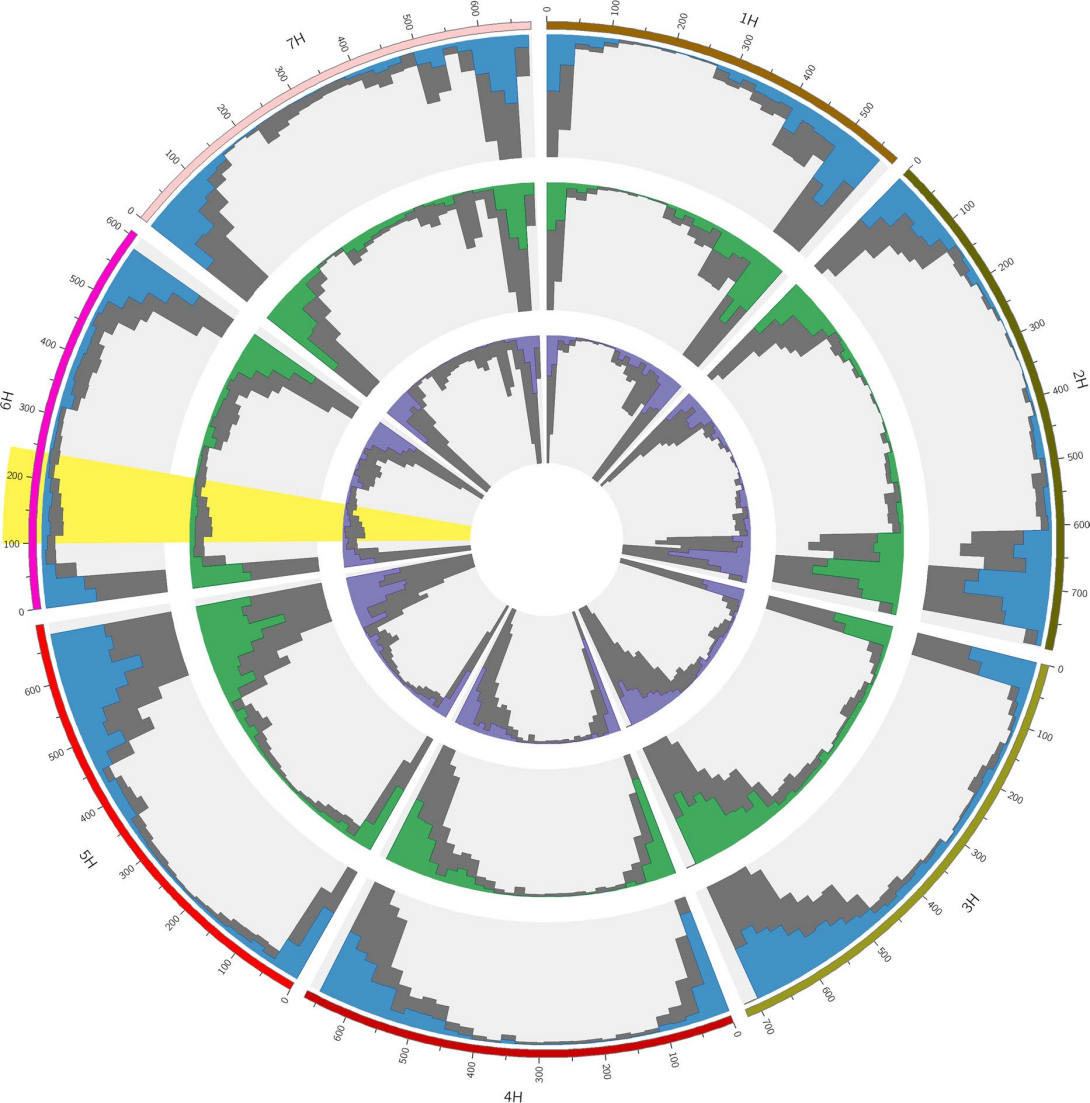
Landscape of the 50 K SNP array marker distribution on seven barley chromosomes. All SNPs from the 50 K Illumina Infinium iSelect genotyping array are presented in grey. SNPs between Chikurin Ibaraki 1 and Igri are presented in blue. SNPs between Chikurin Ibaraki 1 and Uschi are presented in green. Consensus SNPs from the comparison are presented in purple (Chikurin Ibaraki 1 and Igri; Chikurin Ibaraki 1 and Uschi). The interval between flanking markers *rym15*_1 and *rym15*_8 is presented in yellow

The genotyping of three parental lines using the 50 K array identified 14,863 (Chikurin Ibaraki 1 and Igri) and 13,678 (Chikurin Ibaraki 1 and Uschi) polymorphic SNPs (Fig. 1; Supplementary Table 6). In total, 9310 SNPs (68.06%) were identical among parental combinations. On the target chromosome 6H, 1679 (Chikurin Ibaraki 1 and Igri) and 1565 (Chikurin Ibaraki 1 and Uschi) SNPs were identified, of which 1076 SNPs (68.75%) were in common.

**Fig. 2:**
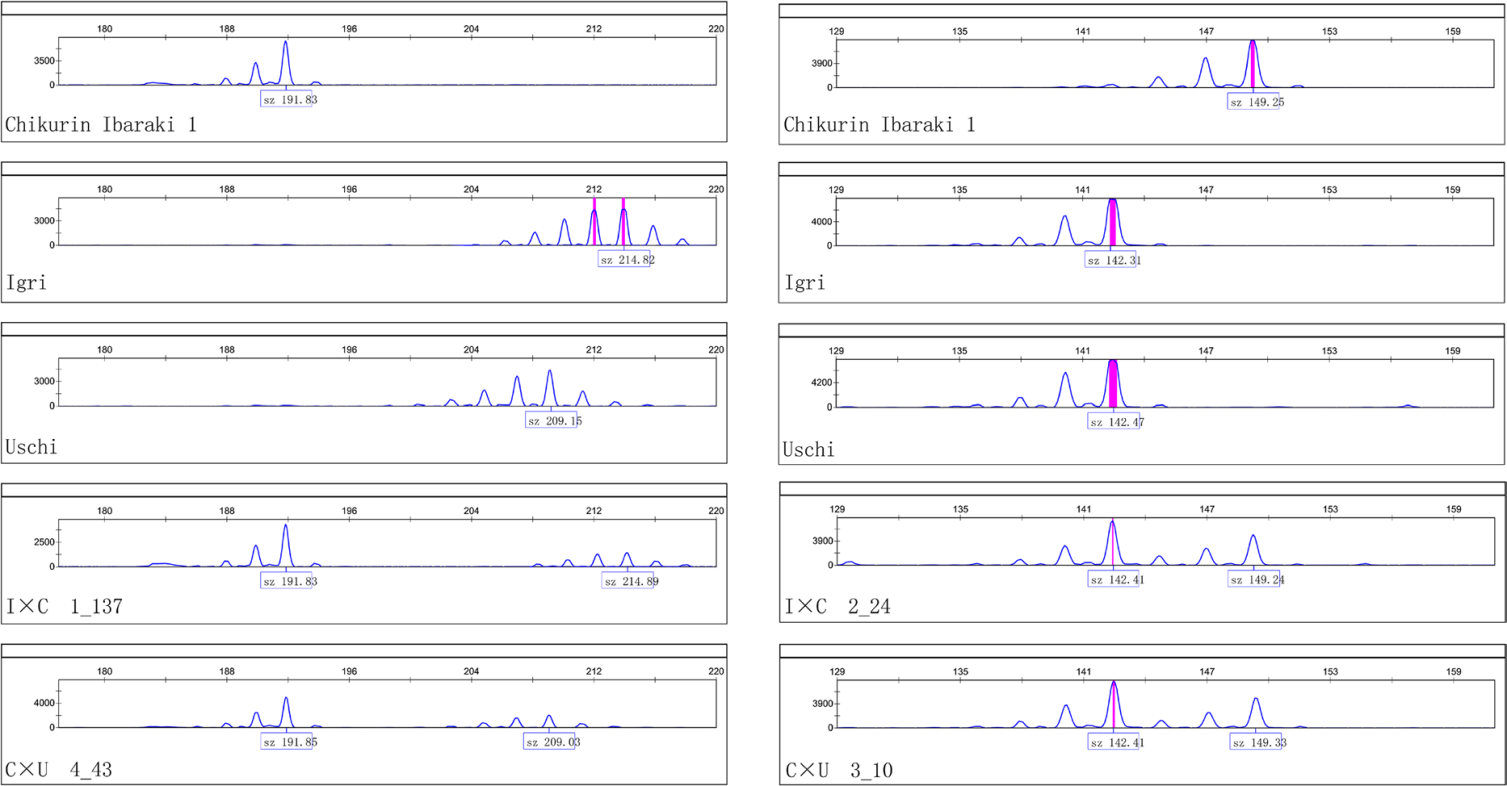
Chromatograms of the SSR markers EBmac0874 (left) and Bmag0173 (right) used for mapping of the resistance gene *rym15*. The order of genotypes for both markers are resistant parent Chikurin Ibaraki 1, susceptible parent Igri, second susceptible parent Uschi, one F_2_-plants from cross Igri × Chikurin Ibaraki 1, and one F_2_-plants from cross Chikurin Ibaraki 1 × Uschi

Three parental lines were genotyped using six SSR and eight KASP markers (Table 2; Fig. 2). The genotypic ratios of all markers in F_2_ families from both the F_2_ populations fitted to a 1:2:1 segregation ratio (Supplementary Table 7). The physical position of all used markers was determined using the blastN alignment algorithm against both publicly available Morex assemblies (Table 2). The two distal, telomeric SSR markers Bmag0173 and Bmag0870 span around 299.78 Mb on chromosome 6H according to the Morex v2 assembly. Controversially, for the SSR marker Bmag0173, no hits on chromosome 6H in Morex v1 could be found, while the blastN alignment of the reverse primer against Morex v2 revealed a hit on chromosome 6H (Supplementary Table 8).

**Table 2:** Physical position and allele size/types of SSR and KASP markers

Marker		Bmag0176	EBmac0874	Bmag0867	Bmac0127	Bmac0018	Bmag0870	*rym15*_1	*rym15*_4	*rym15*_6	*rym15*_8	*rym15*_11	*rym15*_13	*rym15*_15	*rym15*_17
Physical position_Morex v1	Start	-	150,284,733	261,541,306	271,882,721	293,925,242	397,650,916	100,092,059	175,284,342	200,044,740	240,373,116	287,521,970	319,250,935	338,666,322	348,226,696
	End	-	150,284,844	261,541,434	271,182,699	293,925,223	397,651,036	-	-	-	-	-	-	-	-
Physical position_Morex v2	Start	-	148,343,963	258,379,492	272,699,163/272699616	295,207,402	395,517,134	99,216,348	174,152,954	198,364,820	235,707,335	289,171,679	321,032,499	339,011,251	347,643,455
	End	95,736,832	148,344,153	258,379,622	272,699,278/272699733	-	395,517,254	-	-	-	-	-	-	-	-
Allele size/type	Igri	142	214	149	135	156	135	A	A	A	T	A	A	C	T
	Uschi	142	209	144	144	156	122	A	A	A	T	A	A	C	T
	Chikurin Ibaraki 1	149	191	137	137	150	148	G	G	G	C	C	G	G	C

### Medium-resolution map construction

**Fig. 3:**
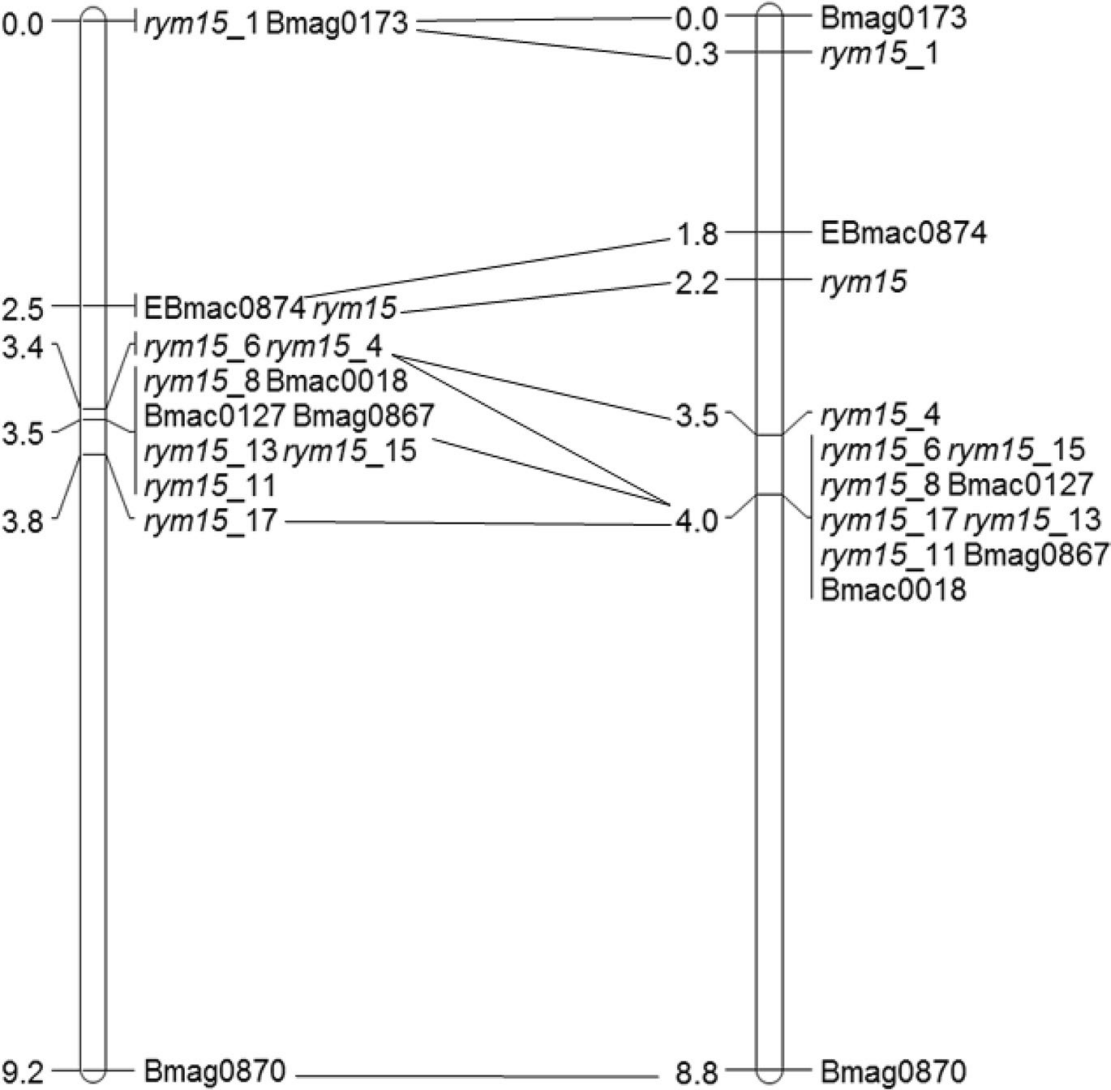
Genetic maps of BaMMV resistance gene *rym15*. Maps were constructed based on analysis of 342 and 180 F_2_ plants derived from the crosses Igri × Chikurin Ibaraki 1 (left) and Chikurin Ibaraki 1 × Uschi (right). Collinearity between the two genetic maps is shown with black lines

The resistance gene *rym15* was mapped between the two flanking markers *rym15*_1 and *rym15*_8 (Supplementary Fig. 1) within a genetic window of 3.5 cm and 3.7 cm in the F_2_ populations I × C and C × U, respectively (Fig. 3). At the same time, the physical size of the interval according to Morex v2 assembly was estimated to be 137 Mb. Between the two flanking markers, 141 and 109 SNPs were identified at the I × C and C × U populations, respectively, of which a set of 85 SNPs (77.98%) was in common.

## Discussion

Following initial genetic mapping of the gene of interest, the next step towards positional isolation of candidate genes is an immediate screening of a large population with dense markers segregating at the locus of interest, commonly referred to as high-resolution mapping. In the present study, due to the non-collinear order of previously published flanking markers (Le Gouis et al. 2004; Ramsay et al. 2000) and the high rate of resistant genotypes that were identified during infection of the first batch of F_2_ plants, we decided to remap the gene at medium resolution in a smaller population to resolve the previous discrepancies. Instead of developing the high-resolution mapping populations by using the previous flanking markers, analysis of 342 (I × C) and 180 (C × U) F_2_ plants was conducted. The main aim of this step was to take into account an actual ratio of hampered phenotyping and to optimize map base cloning by mapping the gene to a smaller interval. The use of KASP markers with a precise position in contrast to the previously flanking SSR markers (Le Gouis et al. 2004), together with the construction of a medium size mapping population, might help optimizing costs and time constrains during map-based cloning.

Since the BaMMV resistance gene *rym15* originates from a non-adapted landrace and is currently not used in breeding programs in Germany, a detailed evaluation of the mechanical inoculation was performed. The ELISA score revealed that 96.35%, 87.5%, and 100% of susceptible control Maris Otter and susceptible parental lines Igri and Uschi, respectively, were infected. The susceptible control cultivar Maris Otter showed a higher rate of infectivity than parental cultivar Igri, corresponding to previous studies (Yang et al. 2014b; Shi et al. 2019). At the same time, parental cultivar Uschi revealed a higher infection rate than Maris Otter, albeit with a lower number of tested plants. In the case of F_2_ and F_3_ plants, the efficiency of the mechanical inoculation varied from 90.56 to 93.23% in the I × C and C × U populations, respectively. In the F_2_ populations, we analyzed a currently used method based on finger rubbing for mechanical inoculation which revealed about 10% escapes. However, the efficiency in the present study was much higher than in similar studies (Shi et al. 2019; Pidon et al. 2020), although a similar inoculation method (Habekuß et al. 2008) was used.

The combined F_2_ and F_2:3_ analysis revealed that a single recessive resistance gene on chromosome 6H named *rym15* conferred the resistance against BaMMV in the Japanese cultivar Chikurin Ibaraki 1. The order of markers in two constructed medium-resolution maps turned out to be collinear, and all mapped markers showed the same order in the genetic map and physical map according to the reference position at the Morex v1 and v2 assemblies (Mascher et al. 2017; Monat et al. 2019). In both constructed maps, the markers EBmac0874 and Bmag0173 are inverted compared to the previous map (Le Gouis et al. 2004); in addition, in the present study, the interval fixed by these two markers is out the frame of the target region containing *rym15* locus. According to the physical position of the reverse primer at the Morex v2 genome assembly, the Bmag0173 revealed to be distal to EBmac0874, which corresponds to the order in the present study. These two markers have been used in several studies, and some maps show the same order as the present study (Ramsay et al. 2000; Varshney et al. 2007; Friesen et al. 2006; Gupta et al. 2011), and some show the discrepancy in the order in comparison with the present study (Cakir et al. 2003a, b; Le Gouis et al. 2004). In addition, the distances of these two markers are very different between the maps, which could be explained by the use of a different type of population, the size of the population, and the differences in the genetic background of the genotypes used. In conclusion, the discrepancy of the SSR markers EBmac0874 and Bmag0173 is commonly known and not unique. A hypothetical explanation of discrepant mapping could be co-migration of fragments from two or more loci in certain genotypes versus presence of single bands in other genotypes.

The high-quality barley reference sequences Morex v1 and Morex v2 (Mascher et al. 2017; Monat et al. 2019) provide more precise information than the draft barley genome sequence (The International Barley Genome Sequencing Consortium, 2012). The study of leaf rust resistance gene *Rph*_*MBR1012*_ (Fazlikhani et al. 2019) has shown the efficient use of the barley reference sequence (Morex v1), especially in marker saturation. In the present study, the SNPs derived from the 50 K Illumina Infinium genotyping array were positioned on the physical map based on the published barley reference sequence. For the previous flanking SSR marker Bmag0173, no information about physical position on chromosome 6H could be found in Morex v1, while based on the Morex v2 assembly, the physical position of the reverse primer provide more precise information; thus, it could be used as reference information when comparing the order of these two previous flanking markers, reflecting the improvement of Morex v2 compared to the Morex v1.

A major constraint in map-based cloning projects is the interplay between the size of the target region defined by flanking markers and the number of F_2_ plants needed for delineation of a single candidate gene. However, barley and other Triticeae are rich in repetitive DNA which hampers gene isolation (Krattinger et al. 2009). Nevertheless, nowadays, based on the reference sequence of Morex, high-throughput genotyping (e.g., via genotyping-by-sequencing or high-density SNP arrays) can considerably improve the efficiency of marker development in barley. In the present study, the KASP markers were developed in a short time based on the screening of parental lines by using 50 K Illumina Infinium genotyping array. The medium-resolution maps we constructed provide more reliable results for delineating the target gene. In case of incorrectly scored phenotypes, this step greatly reduces the risk that a gene of interest may lie outside of putative flanking markers which span a very short interval.

The next step for isolating the resistance gene *rym15* is the construction of a high-resolution map. For this, high-resolution mapping populations will be constructed by screening the newly developed, robust flanking markers in around 8000 F_2_ plants from both F_2_ populations. For marker saturation, a set of 85 informative SNP markers was identified between the flanking markers *rym15*_1 and *rym15*_8 based on the 50 K SNP array screen. Based on information on corresponding candidate genes (high confidence and low confidence) in the genome interval covered by these SNPs, promising genes will be selected for marker development for further saturation of the *rym15* locus. Meanwhile, the KASP markers developed in the present study can already be efficiently used in breeding programs attempting to transfer *rym15* to elite barley cultivars.

## Supplementary Information

Below is the link to the electronic supplementary material.

Supplementary file1 Flanking markers for rym15. a) Observed segregation from the marker rym15_1 in F2 families from the population Igri × Chikurin Ibaraki 1 as illustrated by the distinct clustering of resistance (orange) heterozygote (green) and susceptible (blue). b) Observed segregation from the marker rym15_8 in F2 families from the population Igri × Chikurin Ibaraki 1 as illustrated by the distinct clustering of susceptible (orange) heterozygote (green) and resistance (blue) (TIF 1927 KB)

Supplementary file2 (XLSX 29 KB)

## Data Availability

All data are given in the manuscript; for materials, please contact the corresponding author.
