# Supplementary figures and images for: Delineating the elusive BaMMV resistance gene rym15 in barley by medium-resolution mapping
Source: Mol Breed. 2021 Dec 2;41(12):76. doi: 10.1007/s11032-021-01270-9 (PMC10236098; doi:10.1007/s11032-021-01270-9)

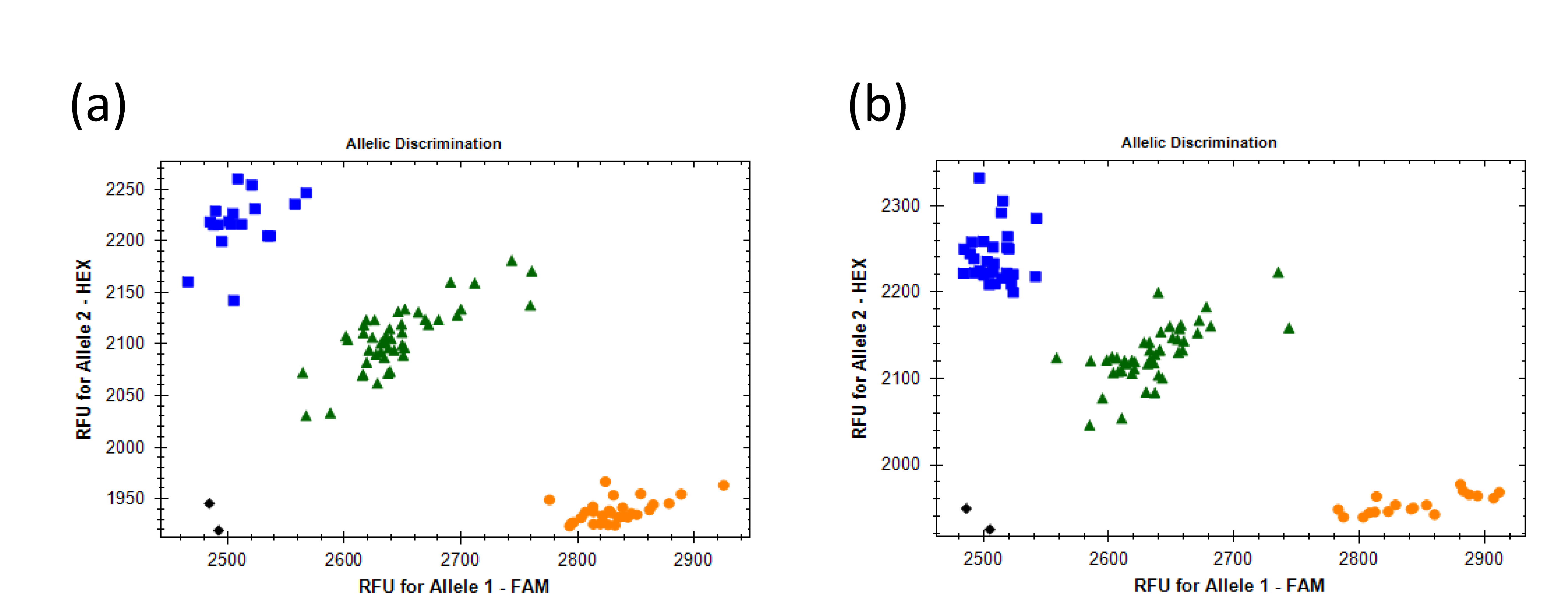

Supplement: Supplementary file 1 — Supplementary file1 Flanking markers for rym15. a) Observed segregation from the marker rym15_1 in F2 families from the population Igri × Chikurin Ibaraki 1 as illustrated by the distinct clustering of resistance (orange) heterozygote (green) and susceptible (blue). b) Observed segregation from the marker rym15_8 in F2 families from the population Igri × Chikurin Ibaraki 1 as illustrated by the distinct clustering of susceptible (orange) heterozygote (green) and resistance (blue) (TIF 1927 KB) [file 11032_2021_1270_MOESM1_ESM.tif]
